# Diagnostic Challenges in a Case of Hemophagocytic Lymphohistiocytosis Likely Induced by Coxiella burnetii

**DOI:** 10.7759/cureus.81634

**Published:** 2025-04-02

**Authors:** Jody W Tai, Phillis Wu

**Affiliations:** 1 Internal Medicine, Olive View University of California Los Angeles Medical Center, Los Angeles, USA; 2 Hematology and Oncology, Olive View University of California Los Angeles Medical Center, Los Angeles, USA

**Keywords:** coxiella burnetti, hemophagocytic lymphohistiocytosis (hlh), hlh-94, h-score, q-fever

## Abstract

Hemophagocytic lymphohistiocytosis (HLH) is a syndrome of pathologic overactivation of the immune system that leads to excessive inflammation and tissue destruction. It can be genetic or acquired in the setting of infection, autoimmune disorders, or malignancy. Here we present a case report of a patient who was initially thought to have severe disseminated intravascular coagulation (DIC) but was ultimately diagnosed with HLH per the 2004 criteria, most likely secondary to *Coxiella *infection.

A 69-year-old female presented with cough, acute hypoxic respiratory failure, and altered mental status. Laboratory findings were significant for coagulopathy initially thought to be DIC, but she was subsequently diagnosed with HLH. Bone marrow biopsy showed increased histiocytes showing hemophagocytosis. The trigger for the HLH was initially unclear, but the patient was eventually found to meet Q fever criteria after being found to be positive for *Coxiella*. She underwent treatment with the HLH-94 protocol, which includes high-dose steroids and etoposide with normalization of hematologic labs and resolution of symptoms. She was also treated with a standard course of doxycycline for *Coxiella*.

HLH should be considered in patients who present with DIC-like labs and other hematologic abnormalities. In such cases, using the H score can assist in raising suspicion for reactive hemophagocytic syndrome. A biopsy of the involved site, such as bone marrow, showing increased hemophagocytosis can also help with confirming the diagnosis. It is important for early identification and initiation of treatment for HLH to improve survival outcomes.

## Introduction

Hemophagocytic lymphohistiocytosis (HLH) is a hyperinflammatory syndrome due to dysregulated immune system activation that leads to host cell phagocytosis and cytokine storm, resulting in multiorgan failure [1]. HLH is most commonly seen in the pediatric population and is usually associated with mutations in various genes involved in cytolysis and intracellular trafficking [2]. However, adult HLH cases are typically triggered by malignancy, autoimmune diseases, infection, or even bone marrow transplant. Up to 68% of cases in patients aged 14-29 years are associated with infection [3]. We present an interesting case of a patient whose HLH was ultimately determined to be triggered by Q fever, a zoonotic infection caused by the gram-negative bacterium *Coxiella burnetii*.

## Case presentation

A 69-year-old female with a past medical history of hypertension presented with cough, myalgias, and dyspnea. She initially went to an outside urgent care three days prior with the same symptoms. At that time, her respiratory viral panel was negative for Coronavirus disease 2019 (COVID-19), influenza, respiratory syncytial virus, or strep and therefore was discharged with supportive care. However, the patient re-presented to urgent care in the setting of worsening weakness, cough, and dysuria. There she was found to be hypoxemic with a pulse oximetry saturation of 86% on room air and was subsequently sent to the emergency room.

Social history was significant for exposure to pet birds, a dog, and a feral cat, which recently gave birth to kittens at the patient’s house. There was also a rat located in the toilet bowl one evening when the patient went to the bathroom, which ran up the shower curtain but did not bite or touch her. She had been vaccinated within the last four months for the influenza virus. Relevant regional information was that the area of presentation, where the patient had been living for decades, is not endemic to Q fever.

Upon presentation to the emergency room, the patient had a temperature of 37.9 degrees Celsius, a heart rate of 114 beats per minute, a blood pressure of 89 mmHg/67 mmHg, and an oxygen saturation of 86% on room air, requiring two liters of nasal cannula to maintain saturation above 95%. The physical exam was notable for lethargy, ecchymoses noted on the bilateral plantar aspects of her feet extending to the toes without cyanosis, with some coldness noted but adequate capillary refill and palpable pulses. She was alert to name only but responded to painful stimuli and was noted to be protecting her airway.

Laboratory investigations included a complete blood count (CBC), white blood cell (WBC), hemoglobin (Hgb), lactate dehydrogenase (LDH), international normalized ratio (INR), aspartate aminotransferase (AST), alanine aminotransferase (ALT), and other relevant tests. These results are summarized in Table 1.

**Table 1:** Laboratory results on admission WBC: white blood cell; Hgb: hemoglobin; LDH: lactate dehydrogenase; INR: international normalized ratio; AST: aspartate aminotransferase; ALT: alanine aminotransferase

Test	Result	Reference range
WBC	7.8 K/cumm	4.5-10.0 K/cumm
Hgb	13.8 g/dL	12.0-14.6 g/dL
Platelets	12 K/cumm	160-360 K/cumm
Creatinine	1.19 mg/dL	0.50-1.00 mg/dL
Alkaline phosphatase	172 U/L	38-126 U/L
AST	226 U/L	15-41 U/L
ALT	92 U/L	14-54 U/L
Total bilirubin	3.5 mg/dL	0.1-1.2 mg/dL
LDH	591 U/L	98-192 U/L
Ferritin	10,003 ng/mL	5-204 ng/mL
Haptoglobin	< 15 mg/dL	36-195 mg/dL
Prothrombin time	16.1 sec	11.5-14.5 sec
INR	1.31	0.86-1.15
D-dimer	> 20 mcg/mL	< 0.49 mcg/mL
Fibrinogen	99 mg/dL	215-450 mg/dL
Triglycerides	584 mg/dL	< 149 mg/dL

**Figure 1:**
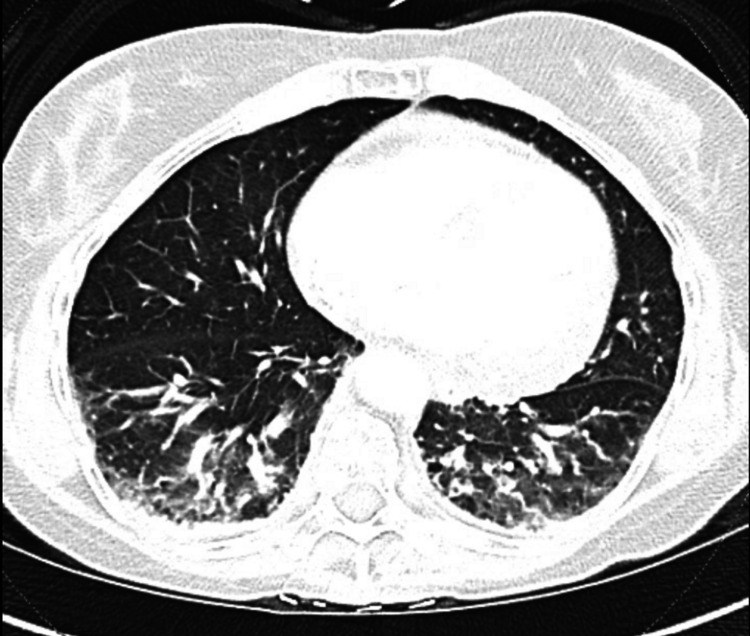
CT pulmonary angiography showing tree-in-bud nodularities CT: computed tomography

Notably, the patient’s platelets had been normal at 239 K/cumm just three weeks prior to presentation. Chest X-ray showed a new mild irregular patchy interstitial and airspace opacification in the lower lung zones. Computed tomography (CT) pulmonary angiography showed a 1.0 cm pericardial effusion with multifocal foci of tree-in-bud nodularities throughout the lungs (Figure 1).

Initial differentials included coagulopathies such as disseminated intravascular coagulation (DIC), thrombotic thrombocytopenic purpura (TTP), or hemolytic uremic syndrome (HUS). There was also concern for infectious etiologies given the pulmonary tree-in-bud nodularities and recent animal exposures. Infectious disease was consulted and performed a workup for *Bartonella*, murine typhus, hantavirus, *Coxiella*, psittacosis, *Mycoplasma pneumoniae*, or leptospirosis. The patient was started on empiric ceftriaxone and doxycycline pending infectious serologies. A bone marrow biopsy was performed, which showed hypercellular marrow with multilineage maturation, serous fat atrophy, and increased histiocytes showing hemophagocytosis. No excess blasts or evidence of lymphoproliferative neoplasm were seen.

**Figure 2:**
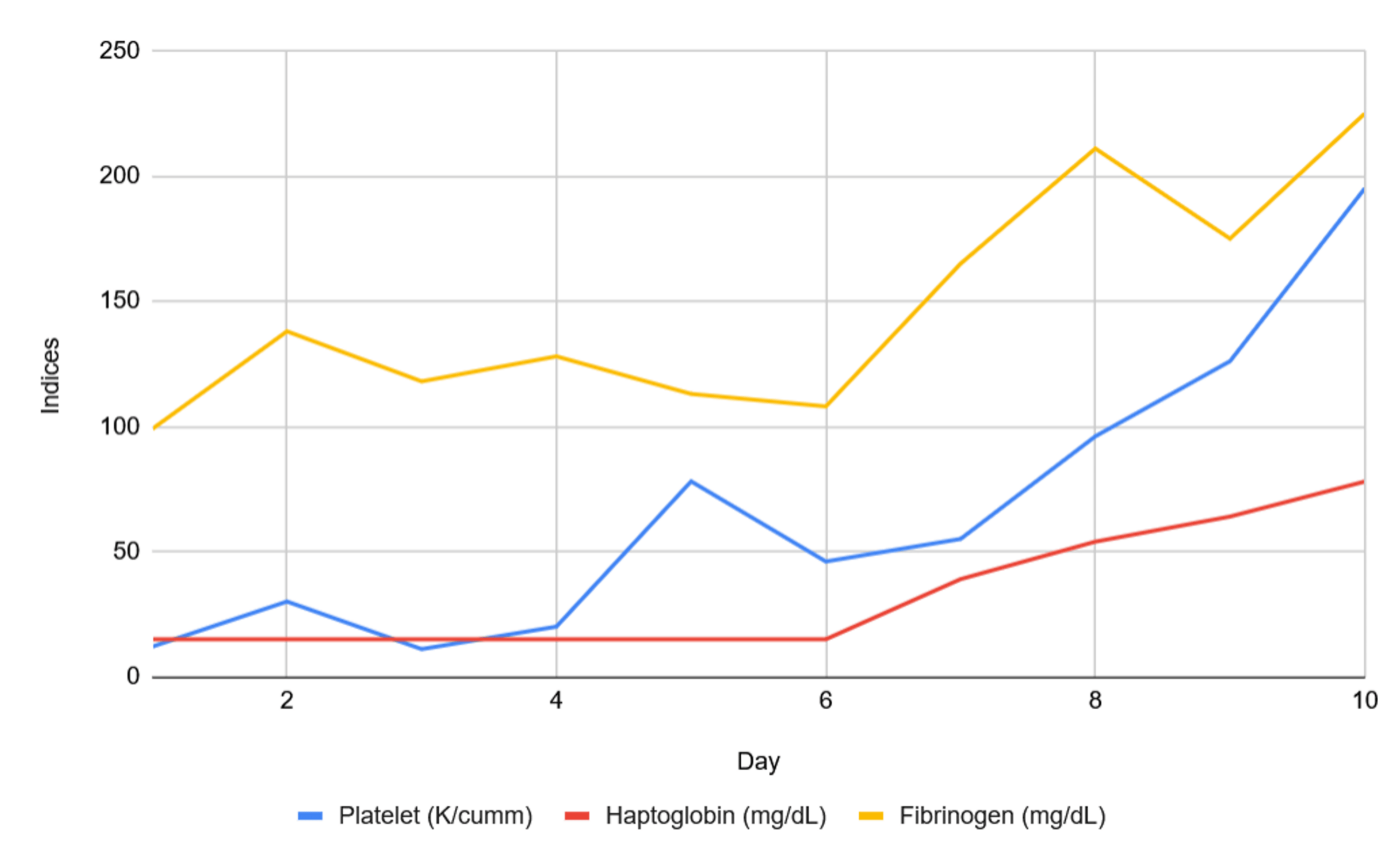
Trend of key hematologic indices

With this collective impression of laboratory results, the top differential became HLH. As such, the patient was started on the HLH-94 protocol with decadron and etoposide. Later in the hospital course, infectious studies were positive for Q fever immunoglobulin G (IgG) phase 2 at 1:16 (negative reference: less than 1:16) and *Rickettsia typhi* immunoglobulin M (IgM) at 1:64 (negative reference: less than 1:64). She was deemed to meet the case classification of probable acute Q fever with headache, acute hepatitis, and pneumonia, which was thought to be the underlying trigger, and completed treatment with 14 days of doxycycline. After discharge, she continued to receive weekly etoposide with an oral dexamethasone taper. The patient completed treatment of the HLH-94 protocol and remains stable in the outpatient setting with normalization of key hematologic indices (Figure 2).

## Discussion

HLH is a hyperinflammatory overactivation of the immune system related to defects in cytotoxic lymphocyte function [4]. HLH is classified as primary and secondary. Primary or hereditary HLH is a result of genetic mutations, most usually in the perforin-1 (PRF1) gene, that leads to overproduction of the pore-forming protein, resulting in destabilization of cells with widespread increase in cytokines [5]. Secondary HLH is defined as HLH presenting in adults secondary to a trigger rather than a true genetic mutation. These triggers can include infection, malignancy, or macrophage activation syndrome in autoimmune disorders [6]. There is no generalized consensus for the prevalence of primary or secondary HLH given overlapping diagnostic features and confounding comorbidities at the time of presentation. For primary HLH, the literature review shows reliance on data collection from 1987 to 2006 in a Swedish national registry that showed a yearly incidence of roughly 1.5 per million [5]. However, for secondary HLH, prevalence relies on reported estimates of 1 in 2000 for critical care admissions in a retrospective review of a single academic tertiary center [7]. Regardless, HLH is significant for an overall mortality as high as 25% [8].

Diagnosis can be challenging at the time of presentation, given multiple comorbidities that mask underlying hematologic abnormalities. For example, the initial differential diagnosis was thought to be DIC in this patient due to the presence of coagulopathy and severe thrombocytopenia. However, the elevated ferritin and appropriate response to transfusions made this less likely. Accordingly, the first major classification for diagnostic criteria is known as HLH-94, a trial that required the following five out of five markers to be met for diagnosis: fever, cytopenia of at least two lineages, splenomegaly, hypertriglyceridemia with or without hypofibrinogenemia, and biopsy-proven hemophagocytosis [5].

Subsequently, with the HLH-2004 trial, an additional three laboratory findings were added, and an established diagnosis was determined to be at least five out of the combined eight criteria: ferritin greater than 500 ng/ml, low or absent NK-cell activity, and elevated soluble form of the interleukin-2 receptor (sIL2Ra) levels greater than or equal to 2400 U/ml. Notably, these five out of eight criteria do not necessarily need to include biopsy-proven hemophagocytosis. Despite these guidelines, it can still be difficult to confirm the diagnosis since the criteria can be nonspecific and need to be interpreted in the overall clinical context. Furthermore, bone marrow biopsy results and sIL2Ra evaluation may not be easily accessible at all hospitals. This subsequently led to the creation of the HScore, a logarithmic calculator developed in a multicenter retrospective cohort of adults to help clinicians estimate the probability of hemophagocytic syndrome and guide empiric treatment decisions. Further studies of the HScore tool showed high sensitivity and specificity of HScore in pediatric (100%, 89.9%), adult (92.9%, 100%), and the entire population (92.86%, 95.45%) when compared to the 2004 guidelines [9].

Treatment for adult cases includes treating the underlying condition causing HLH as well as consideration of the HLH-94 protocol, a combination of chemotherapy and steroids as an extrapolation of pediatric HLH regimens. This involves corticosteroids, usually dexamethasone starting at 10 milligrams per meter squared and tapered down, and etoposide, a DNA synthesis inhibitor selected for specificity against cytokine and T-cell proliferation, dosed at 150 milligrams per meter squared [10]. Intrathecal therapy can be considered if there are abnormal cerebrospinal fluid (CSF) findings or progressive neurologic symptoms.

In the case of this specific patient, she received dose-adjusted etoposide due to elevated liver enzyme tests and acute kidney injury. The HLH-94 treatment protocol was selected based on institutional protocol. Fortunately, our patient showed signs of clinical improvement; refractory cases often involve treatment with T-cell depleting agents such as alemtuzumab or emapalumab, which are often used as bridges to stem cell transplant [11]. There was no evidence of central nervous system involvement with normal CT of the brain and negative CSF pathology. Ultimately, the trigger of this patient’s secondary HLH was not entirely conclusive. Although she met clinical evidence of Q fever, the *Coxiella *IgG phase two positivity was in the supportive but not diagnostic range. This presumption of probable acute Q fever was based on Q Fever Working Group criteria [12]. The diagnosis could have been confirmed by repeating the phase two IgG antibody titer to evaluate for a fourfold rise between the acute and convalescent setting; unfortunately, this was not tested. It was also unclear if the positive *R. typhi* IgM represented cross-reactivity in the setting of possible Q fever. Regardless, she showed improvement with a standard 14-day doxycycline treatment. Notably, in our literature review, there has only been one other case that was published in 2020 of HLH induced specifically by *C. burnetii* [13].

## Conclusions

HLH is a hyperinflammatory immune response with a high overall mortality rate and should be managed with urgent interdisciplinary care, which is critical for achieving favorable outcomes. Clinicians should be aware of its underlying features with other high-risk coagulopathies such as DIC and recognize the complexity of diagnosing both HLH and its triggers. Further investigation is required to determine the optimal selection of treatment between the two most common protocols of HLH-94 versus HLH-2004.
